# Like Visiting an Old Friend: Fischer Glycosylation in the Twenty-First Century: Modern Methods and Techniques

**DOI:** 10.1007/s41061-022-00383-9

**Published:** 2022-05-21

**Authors:** Matteo Haese, Kai Winterhalter, Jessica Jung, Magnus S. Schmidt

**Affiliations:** Institute of Precision Medicine, Organic and Bioorganic Chemistry Labs, Medical and Life Sciences Faculty, Furtwangen University, Jakob-Kienzle-Str. 17, 78054 VS-Schwenningen, Germany

**Keywords:** Fischer glycosylation, Glycosides, Microwave, Ultrasonic, Microreactor, Flow reactor

## Abstract

Fischer glycosylation is typically the chemical reaction of a monosaccharide and an alcohol in presence of an acidic catalyst to afford glycosides in pyranosidic and furanosidic forms. This reaction is still applied today for the synthesis of specialized glycosides, and optimization and modification of the method have continued since its discovery by Emil Fischer in the 1890s. This review presents advancements in Fischer glycosylation described in literature of the past 15 years and its implementation in modern chemical methods.

## Introduction

Carbohydrates play a major role in the development and functionality of organisms. For many years, research exploring carbohydrates was regarded as less interesting than most other topics in biochemistry. However, with time, this view has changed and the vital role that carbohydrates play in many processes is attracting more research interest [1]. Due to the heightened interest in glycoscience, the importance of glycosides in many biological processes has also come to light. This shows a clear need for strategies to synthesize specific glycosides. Significant effort has been invested in research into various synthetic strategies such as the Koenigs–Knorr reaction, the more versatile trichloracetimidate method, as well as others [2–8]. Despite the number of different protocols reported, the approach first mentioned and developed in the 1890s by the German chemist Emil Fischer remains a very valuable method for synthesis of simple glycosides. In the classical approach described by Fischer, the sugar is dissolved in an alcohol in the presence of a strong acid. It is known that, owing to the presence of water as a byproduct, the formed oxocarbenium ion can react back to the reducing sugar. This unwanted effect can be addressed by running the reaction in an excess of alcohol to shift the equilibrium towards the desired end product. The reaction delivers a mixture of furanosides and pyranosides, with furanosides being the product of kinetic control while the more stable pyranosides can be derived through thermodynamic control. The basic mechanism of Fischer glycosylation is described throughout the scientific and educational literature [9,10].

While the main benefit of Fischer glycosylation lies in its simplicity, the approach suffers from limitations such as the need for strong Lewis acids and long reaction times, as well as the production of a mixture of products of furanosides and pyranosides as well as their anomeric forms [11,12]. These shortcomings show the need for new approaches towards Fischer glycosylation to overcome these limitations. In this review, we look at different approaches such as the use of new catalysts and technologies such as microwave or flow chemistry.

## Use of Different Catalysts and Additives for Fischer Glycosylation

Several reagents have been successfully implemented as new catalysts for Fischer glycosylation. While increasing product yield and selectivity was generally a desired effect, catalysts have also been chosen on the basis of their ecofriendliness and ability to synthesize sugars, without the need for protecting groups. Modifications of the classic Fischer glycosylation method have also been applied, for example, adding organic additives to the reaction to increase yield or creating a micellar reaction system to increase the solubility of carbohydrates in alcohols.

### Sulfuric Acid Immobilized on Silica for Synthesis of Glycosides from Free Sugars

**Fig. 1:**
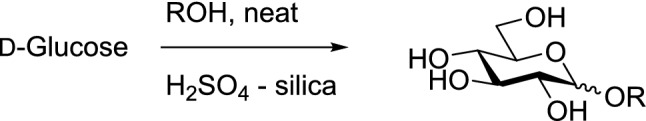
General scheme for synthesis of glycosides catalyzed by H_2_SO_4_-silica

Sulfuric acid immobilized on silica (H_2_SO_4_-silica) is a catalyst that was used by Roy et al. to shorten the reaction time and reduce the amount of alcohol required to prepare glycosides from free sugars by Fischer glycosylation (Fig. 1) [13].

In a first approach, the authors added d-glucose and H_2_SO_4_-silica to propargyl alcohol at 65 °C and observed the reaction over time. After cooling to room temperature, excess propargyl alcohol was eluted with dichloromethane (CH_2_Cl_2_) over a silica gel column, followed by elution of the product with a dichloromethane–methanol (CH_2_Cl_2_–MeOH) (15:1) mixture, providing the desired propargyl glycoside. The product was per-*O*-acetylated with Ac_2_O and H_2_SO_4_-silica for ^1^H and ^13^C nuclear magnetic resonance (NMR) analysis (Table 1) [13].

**Table 1:** Comparison between anomeric selectivities obtained by different methods [13]

Method	α:β	Yield (%)
Method A: 2.5 h stirring at 65 °C (until complete dissolution of starting material)	6:1	75
Method B: after 2.5 h heating at 65 °C, the mixture was allowed to stir at room temperature for 12 h	10:1	81
Method C: stirring continued at 65 °C for an additional 3 h after complete dissolution	10:1	80

No formation of furanosides or acyclic acetals as byproducts has been observed. Method C provided good anomeric selectivity and good yields in a short time compared with methods A and B. Using method C, several propargyl glycosides were prepared with different reducing sugars (Table 2) [13].

**Table 2:** Preparation of propargyl glycosides from free sugars^a^ [13]

Sugar	Product	Time (h)	Yield (%)	α:β
d-Glucose		5.5	80	10:1
d-Galactose		6	79	10:1
d-Mannose		2	83	1:0
*N*-Acetyl-d-glucosamine		2	80	1:0
l-Rhamnose		2	82	1:0
l-Fucose		4	74	12:1
d-Maltose^b^		8	69	11:1

^a^1 mmol sugar was reacted with 5 mmol propargyl alcohol and 5 mg H_2_SO_4_–silica

^b^For maltose, 10 mmol propargyl alcohol was used for 1 mmol sugar

The reaction yielded glycosides between 69% and 83% with α-isomer-favored selectivity. Using the same method, the reaction was performed with d-glucose and various alcohols (Table 3).

**Table 3:** Preparation of glycosides of d-glucose with different alcohols [13]

Alcohol	Product	Time (h)	Yield (%)	α:β
Allyl alcohol		6	79	10:1
Benzyl alcohol		7	78	10:1
*p*-Methoxybenzyl alcohol		7	75	11:1
*n*-Octanol		6	81	13:1
*n*-Dodecanol		8	76	12:1
2-Bromoethanol		6	82	11:1

The reaction yielded glycosides between 75% and 82%, and again the α-isomer was the favored anomer. The strategy provided by the authors is applicable for large-scale preparations, and the purification of the product only requires filtration [13].

### Acid Zeolites for Glycosylation of *N*-Acetylgalactosamine

**Fig. 2:**
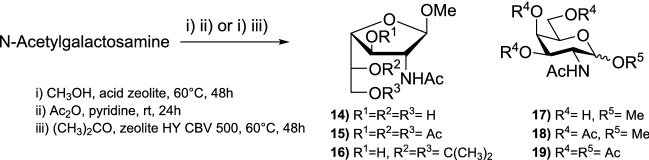
Fischer glycosylation of GalNAc with methanol catalyzed by acid zeolites

Rauter et al. used HY, HZSM-5, and HBEA acid zeolites as catalysts for glycosylation of *N*-acetylgalactosamine (GalNAc) to β-galactofuranosides and β-galactopyranosides. Acidic zeolites are solid, ecofriendly catalytic materials that can differentiate molecules based on their size and form because of their pore and zeolite channels [14]. Within the pores, high selectivity and product concentrations can be achieved. For glycosylation of GalNAc with methanol, four acid zeolites with different characteristics were examined (Fig. 2, Table 4) [15].

**Table 4:** Characteristics of the HY, HZSM-5, and HBEA zeolites [15]

Zeolite	Si/Al ratio	External surface area (m^2^ g^−1^)	Pore volume (cm^3^ g^−1^)^a^		Acid site concentration (µmol g^−1^)	
			Micropore	Mesopore	Brønsted	Lewis
HY (3.1)	3.1	37	0.30	0.05	670	247
HY (13.2)	13.2	64	0.33	0.11	327	74
HZSM-5 (13.3)	13.3	4	0.17	0.03	469	102
HBEA (12.5)	12.5	178	0.19	0.38	315	340

^a^Micropore (Ø < 2 nm); mesopore (2 nm < Ø < 50 nm)

For the reaction, activated zeolite was added to GalNAc in dry methanol and the mixture was refluxed under stirring for 48 h at 60 °C. After filtering the zeolite and evaporating the solvent, the residue was dissolved in pyridine and acetic anhydride was added. The mixture was stirred for 24 h at room temperature. After work-up, compounds **15** and **18** could be isolated in different ratios (Table 5) [15].

**Table 5:** Yield (%) of Fischer glycosylation of GalNAc with methanol followed by acetylation, mediated by acid zeolites [15]

Zeolite	Fischer glycosylation yield (%)		
	15	18	Recovered starting material
HY (3.1)	67	31	2
HY (13.2)	32	14	52
HZSM-5 (13.3)	28	2	69
HBEA (12.5)	33	15	52

Derivative **15** was found to be favored for all used catalysts. The regioselectivity for the furanoside/pyranoside form was 2:1 for the large-pore zeolites (HY and HBEA) and 14:1 for the medium-pore zeolite (HZSM-5). HY (3.1) yielded the highest amount of product, with only 2% of starting material being recovered. The group suggested that the high concentration of Brønsted acid sites and the higher aluminum content, which leads to higher hydrophilicity, are the reasons for the high efficiency of this catalyst. Using HY (3.1) as catalyst, methyl glycosylation of GalNAc, followed by in situ acetonation, was achieved, which resulted in **16** as the major product in 43% yield. This confirmed that the zeolite increases the selectivity for synthesis of a furanoside form [15].

### Ionic-Liquid-Promoted Fischer Glycosylation

In 2009, Jaques Auge et al. reported the use of ionic liquids together with catalytic amounts of various triflate-salt-based Lewis acids as a system for Fischer glycosylation of several monosaccharides with a variety of shorter and longer alcohols [16]. In their work, they could show that, when using ionic liquids based on 1-butyl-3-methylimidazolium, 1,3-dimethylimidazolium, and 1,2,3-trimethylimidazolium cations with a variety of different anions, the performance of the Fischer glycosylation could be increased significantly compared with reactions in neat alcohols. Additionally, the amount of alcohol could be reduced down to 1 equiv. and the amount of Lewis acid could be reduced down to 1 mol.%, while the yields could be doubled in some cases compared with classical Fischer glycosylation in neat alcohols (high excess) using 5 mol.% Lewis acids.

Another advantage mentioned by those authors was the reusability of the ionic liquids without influencing the reaction performance (at least three cycles), making this process more ecofriendly than other state-of-the-art Fischer glycosylation reactions.

### Sulfamic Acid for Synthesis of Glycosides from Unprotected Sugars

**Fig. 3:**
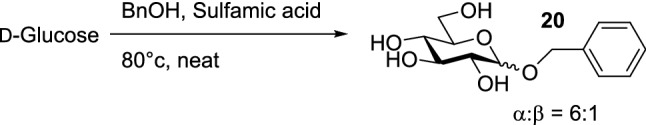
Sulfamic-acid-catalyzed Fischer glycosylation of glucose with benzyl alcohol

In search of an ecofriendly catalyst for Fischer glycosylation of unprotected sugars, Guchhait et al. successfully used sulfamic acid (H_3_NSO_3_) to prepare alkyl glycosides. Sulfamic acid is inexpensive, nonvolatile, noncorrosive, and moderately acidic (p*K*_a_ = 1.0), which prevents the decomposition of the product. The mentioned research group confirmed the catalytic activity of sulfamic acid by the reaction of d-glucose with different quantities of benzyl alcohol in presence of sulfamic acid at 80 °C for 5 h under neat conditions, which led to the formation of an anomeric mixture (α:β = 6:1) of **20** in 81% yield (Fig. 3) [17].

After removal of the solvent, the pure compound was obtained by passing the crude product through a pad of silica. In preparation for ^1^H and ^13^C NMR analysis, the compound was acetylated. After these first results, five unprotected monosaccharides (d-glucose, d-mannose, d-galactose, *N*-acetyl-d-glucosamine, and l-rhamnose) were treated with several aliphatic alcohols under similar reaction conditions. The resulting alkyl glycosides were obtained in satisfactory yield (70–85%), and the major isomer in all cases was the α-isomer. The reaction of sugars with primary and secondary alcohols resulted in alkyl glycosides as products, but with tertiary alcohols no product was formed, which may be because of the polymerization of tertiary alcohols under acidic conditions. A selection of results of these experiments are presented in Table 6 [17].

**Table 6:** Selection of prepared alkyl glycosides by sulfamic-acid-catalyzed Fischer glycosylation of unprotected reducing sugars [17]

No.	Unprotected sugar	Alcohol	Product	Time (h)	Yield (%)	α:β
**1**	d-Glucose	1-Butanol		4.0	85	4:1
**2**		2-Octanol		7.0	70	5:1
**3**		*Tert*-Butanol	No Product	9.0	–	–
**4**	d-Mannose	1-Dodecanol		6.0	70	1:0
**5**		2-Propanol		3.0	82	9:1
**6**	d-Galactose	Propargyl alcohol		4.0	78	8:1
		1-Octanol		4.0	76	6:1
**7**	l-Rhamnose	Allyl alcohol		2.5	85	1:0
		Propargyl alcohol		2.5	80	1:0
**8**	*N*-Acetyl-d-glucosamine	Allyl alcohol		3.0	74	4:1

Additionally, different catalysts for the reaction with d-glucose and benzyl alcohol were compared (Table 7).

**Table 7:** Comparison of catalytic potential of different catalysts in Fischer glycosylation of d-glucose using benzyl alcohol at 80 °C [17]

No.	Catalyst	Catalyst load	Time (h)	Yield (%)
**1**	BF_3_·OEt_2_	1.0 equiv	16	52
**2**	Amberlite IR 120	100 mg/mmol	24	60
**3**	Sc(OTf)_3_	0.1 equiv	24	55
**4**	HClO_4_–SiO_2_	50 mg/mmol	5	72
**5**	Sulfamic acid	0.2 equiv	5	81
**6**	TfOH	0.2 equiv	5	77

Triflic acid (CF_3_SO_3_H) yielded a similar amount of product as sulfamic acid in the same amount of time but is corrosive and moisture sensitive, thus sulfamic acid has been shown to be the favorable catalyst for these kinds of reactions.

### Synthesis of α-Linked Mannobioses with Hydrochloric-Acid-Assisted Fischer-Type Glycosylation

**Fig. 4:**
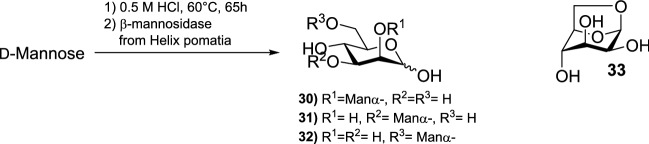
Reverse hydrolysis reaction of d-mannose with water assisted by HCl

Ajisaka et al. developed an acid-assisted reverse hydrolysis reaction based on Fischer glycosylation for synthesis of α-linked mannobioses to replace the more expensive and less efficient enzyme-assisted reverse hydrolysis reaction (Fig. 4) [18].

The alcoholic solution, which is typical for Fischer glycosylation, was replaced with aqueous solution with a very high concentration (83% w/w) of d-mannose to shift the equilibrium towards hydrolysis. To a solution containing 5 g d-mannose and 1 mL water, 240 μL 12.5 M HCl was added to achieve a final concentration of 0.5 M HCl. The mixture was stirred for 65 h at 60 °C. Then, the solution was diluted with water and neutralized with 0.1 M NaOH. To hydrolyze the β-linkages, β-mannosidase was added, and the solution was incubated at 37 °C for 24 h. The mannobioses were separated by activated carbon column chromatography, and their structures were identified by ^13^C NMR spectroscopy. Amounts and yields of the separated products are summarized in Table 8 [18].

**Table 8:** Amounts and yields of isolated products from acid-catalyzed reverse hydrolysis reaction for synthesis of α-mannobioses [18]

No.	Product	Isolated amount (mg)	Yield^a^ (%)
**1**	d-Mannose (recovered)	2900	
**2**	30	166	7.9
**3**	31	167	7.9
**4**	32	614	29.1
**5**	33	14	0.7
**6**	Higher oligosaccharides	158	

^a^Calculated on the basis of the consumed d-mannose

Three different kinds of mannobioses (table entries 2–4) were obtained in moderate yield (45% in total) in one reaction. Compound **33** was a by-product of the reaction. The reaction is based on an equilibrium between mono-, di-, and higher oligosaccharides and can be carried out repeatedly to create an efficient reaction system for the preparation of α-linked mannobioses [18].

### Furandicarboxylic Acid and Its Ester as Organic Additive for Synthesis of Alkyl Polyglucosides

Van Es et al. also used sulfuric acid as catalyst for Fischer glycosylation of d-glucose in decanol to prepare alkyl polyglucosides (APG), specifically decyl glucosides, for cosmetic use and as surfactants. They used furandicarboxylic acid (FDCA) and its mono-*n*-decyl-ester (C10-FDCA) as environmentally friendly, organic additives to increase product yield and limit side reactions to reduce the coloration of the reaction, therefore reducing the requirement for exhaustive purification [19].

For the glycosylation, d-glucose was added to a solution containing decanol and sulfuric acid. The mixture was heated at 95 °C and 50 mbar for 2 h. The crude product was cooled to 80 °C, and sodium carbonate (Na_2_CO_3_) was added at atmospheric pressure. The resulting medium was analyzed by gas chromatography (GC). The reaction was carried out without additives as reference, and the effect of several additives on the reaction was compared (Table 9) [19].

**Table 9:** Glycosylation of d-glucose in decanol catalyzed by sulfuric acid with various organic acid additives [19]

Entry	Additive (mol.%^b^)	Optical density 420 nm	GC yield^b^ (%) of **34**	Residual d-glucose (%)
**1**	–	0.525	30	–^a^
**2**	FDCA (5%)	0.078	58	2
**3**	FDCA (2.5%)	0.057	59	3
**4**	Citric (5%)	0.037	38	–^a^
**5**	*i*-Phthalic (5%)	1.400	38	–^a^
**6**	C10-FDCA (5%)	0.098	54	0
**7**	C10-FDCA (2.5%)	0.016	52	0

Amount of sulfuric acid was 0.9 mol.% based on d-glucose; time 2 h

^a^Glucose analysis was impossible because the residual amount was out of the range of the standardized GC method

^b^Based on d-glucose

Adding FDCA at 2.5 mol.% had the highest positive impact on the yield, increasing it to 59% and lowering the optical density (in this case a degree for the degradation of glucose during the reaction by measuring the coloration) to 0.057 compared with the reference reaction. C10-FDCA at 2.5 mol.% had the highest positive impact on optical density, lowering it to 0.016 at 52% yield. Citric and isophthalic acid only yielded 38% product, and while citric acid lowered the optical density to 0.037, isophthalic acid increased it to 1.4, making these additives less favorable than those with FDCA and C10-FDCA [19].

The polyglucoside mixtures obtained by the reactions with FDCA and C10-FDCA as additives at 2.5 mol.% and 5 mol.% were compared with an *n*-octyl/*n*-decyl polyglucoside product that is often used in detergent industry, based on several physiochemical properties that are indicators for the performance of surfactants (Table 10) [19].

**Table 10:** Surface properties, foaming, and wetting power of glycoside composition [19]

Surfactant composition	CMC (mg L^−1^)	γCMC (mN m^−1^)	Foam volume at *t* = 0 (mL) [stability (%)]	Wetting time (s)
Table 9, entry 1	500	28	450 [62]	31
Table 9, entry 2	424	28	450 [80]	47
Table 9, entry 3	483	28	450 [75]	31
Table 9, entry 6	614	28	430 [62]	33
Table 9, entry 7	596	28	460 [60]	26
*n*-Octyl/*n*-decyl Polyglucosides	963	26	450 [75]	196

The surface tension at critical micelle concentration (CMC) was the same for all glycoside mixtures (28 mN m^−1^) except for the octyl/decyl polyglucosides (26 mN m^−1^). The CMC of the glucosides obtained by adding FDCA were slightly lower (424 mg/L and 483 mg/L) than the CMC of the glucosides obtained without additives (500 mg/L) and much lower compared with the CMC of the octyl/decyl polyglucosides (963 mg/L). The CMC of glucosides obtained by adding C10-FDCA were slightly higher (614 mg/L and 596 mg/L) than the CMC of glucosides obtained without additives. A lower CMC means that a lower concentration of glycosides is needed for an efficient detergent, which is desirable for a more economic process. The foam volume varies slightly between the glucoside mixtures (430–460 mL), but using FDCA as additive increases the foam stability to 80%, while using C10-FDCA decreases it to 60%. Low foam volume and stability are favorable for detergents but unfavorable for cosmetic application. The wetting times of the glycoside mixtures prepared by the authors were all much lower (26–47 s) than in the case of the industrial octyl/decyl polyglucosides (196 s) [19].

### Ammonium Chloride for Synthesis of Alkyl Glycosides

Sharma et al. used ammonium chloride (NH_4_Cl) as catalyst to create glycosides of unprotected monosaccharides and sugar acids under solvent-free conditions with different alcohols. Additionally, the group analyzed the immunomodulatory activity of the products, to find out whether they could potentially be used as adjuvants for vaccines. For the reaction, NH_4_Cl was added to a solution of d-glucose and decanol, and the mixture was heated at 90 °C for 6 h. After completion of the reaction, the mixture was filtered through a silica pad with 0–15% methanol (MeOH) in dichloromethane (CH_2_Cl_2_) to obtain **34** as product [20].

The structure and anomeric ratio of the products were identified by ^1^H and ^13^C NMR spectroscopy. The reaction showed excellent yield (72%), with **34**α as the preferred derivative (α:β = 2.7:1). The reaction was quicker and more efficient compared with other reaction conditions (Table 11) [20].

**Table 11:** Comparison of catalytic potential of different catalysts in Fischer glycosylation of d-glucose using a long-chain alcohol [16,17,20]

No.	Catalyst^a^	Time (h)	Yield^b^ (%)	(α:β)^c^
**1**	Amberlite IR120H resin (100 mg mmol^−1^)	24	50	(4:1)
**2**	InCl_3_ (5 mol%)	24	5	(Nd)
**3**	In(OTf)_3_ (5 mol%)	24	21	(1.5:1)
**4**	Sc(OTf)_3_ (5 mol%)	24	44	(2:1)
**5**	HCl	24	45	(1.6:1)
**6**	NH_4_Cl (1.0 equiv.)	6	72	(2.7:1)

*Nd* not determined

^a^Catalyst loading

^b^Isolated yield

^c^Determined from ^1^H NMR

NH_4_Cl was then used to catalyze the glycosylation of different reducing sugars (donors) with decanol and additionally for the glycosylation of d-glucose with several alcohols (acceptors) with different chain lengths, under the reaction conditions described above (Table 12).

**Table 12:** NH_4_Cl-mediated reaction of donors and acceptors under standardized reaction conditions [20]

No.	Acceptor (2 equiv.)	Donor (1 equiv.)	Product^a^	Yield^b^ (%) (α:β)^c^
**1**	Decanol	d-Glucose	**34**	72 (2.7: 1)
**2**	Decanol	d-Mannose		68 (1: 0)
**3**	Decanol	d-Xylose		68 (1.2: 1)
**4**	Decanol	*N*-Acetylglucosamine		65 (3.4: 1)
**5**	Allyl alcohol	d-Glucose		65 (1.5: 1)
**6**	Propargyl alcohol	d-Glucose		69 (1.5: 1)
**7**	Butyl alcohol	d-Glucose	**21**	70 (2.5: 1)
**8**	Cyclohexanol	d-Glucose		66 (2.3: 1)
**9**	Benzyl alcohol	d-Glucose	**20**	68 (2.3: 1)

^a^Characterized from ^1^H-NMR and ^13^C-NMR

^b^Isolated yield

^c^Determined from ^1^H NMR

The glucosides in Table 12 had good to excellent yield (65–72%) with high anomeric selectivity, and the glycosylation of d-mannose resulted only in α-isomers.

d-Glucuronic acid and *N*-acetylneuraminic acid were analyzed under the same conditions to see whether their corresponding glycosides were formed (Table 13).

**Table 13:** NH_4_Cl-mediated reaction of sugar acids with alcohols [20]

No.	Donor	Acceptor	Product	Yield^a^ (%) (α:β)^b^
**1**	d-Glucuronic acid	Decanol		64 (7:2)
**2**	d-Glucuronic acid	Propargyl alcohol		61 (1.5:1)

^a^Isolated yield

^b^Determined from ^1^H NMR

d-Glucuronic acid reacted well with decanol and propargyl alcohol, and the corresponding glycosides **41** and **42** were formed in good yield and anomeric selectivity. The reaction of *N*-acetylneuraminic acid yielded only an esterified product, instead of its corresponding glycoside, which has not been fully characterized.

### Bismuth Nitrate Pentahydrate for Synthesis of Glycosides from Unprotected and Unactivated Sugars

**Fig. 5:**
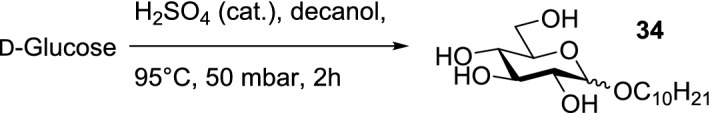
Glycosylation of d-glucose in decanol (decyl oligosides and furanosides omitted for clarity)

Polanki et al. proposed bismuth nitrate pentahydrate (Bi(NO_3_)_3_·5H_2_O) as another ecofriendly catalyst for Fischer glycosylation of unprotected and inactivated sugars. Bismuth nitrate is very accessible and inexpensive and has low toxicity [21]. In preliminary experiments, bismuth nitrate was added to a solution of d-glucose and propargyl alcohol and the mixture was stirred at 60 °C under nitrogen until completion as determined by thin-layer chromatography (TLC). Excess alcohol was removed under vacuum, and the crude product was eluted with a dichloromethane–methanol mixture in a short bed of celite to provide the desired glycoside as an anomeric mixture (α:β = 10:1) in 83% yield. ^1^H NMR spectroscopy was carried out with acetylated crude product to obtain the ratio of α- and β-anomers (Fig. 5) [22].

**Fig. 6:**
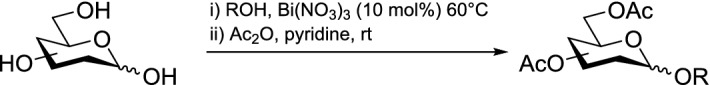
General scheme for synthesis of aryl/alkyl glycosides

The reaction (Fig. 6) was carried out with several other sugars and alcohols under the same conditions to prepare a series of different glycosides. Table 14 shows selected results of the reaction series.

**Table 14:** Selection of aryl/alkyl glycosides obtained by bismuth nitrate pentahydrate-catalyzed Fischer glycosylation of different alcohols and unprotected and unactivated sugars [22]

Donor	Acceptor	Product
d-Glucose	Propargyl alcohol	1
	Allyl alcohol	8
	Benzyl alcohol	9
d-Galactose	Propargyl alcohol	2
	Benzyl alcohol	
l-Rhamnose	Propargyl alcohol	**5**
	Cyclohexanol	
d-Arabinose	Ethanol	
	Cyclohexanol	
*N*-Acetyl-d-glucosamine	Methanol	

The products were obtained in good to excellent yield and anomeric selectivity, with the α-anomer being predominant in all cases. The reaction method was compared with other previously reported methods for Fischer glycosylation based on temperature, reaction time, yield, and anomeric ratio (Table 15) [13,17,22,23].

**Table 15:** Comparison of Bi(NO_3_)_3_·5H_2_O with other methods for Fischer-type glycosylation [13,17,22,23]

Glycoside	Catalyst	Temp. (°C)	Time (h)	Yield^a^ (%)	α:β ratio
**1**	Sulfamic acid	80	4	78^[[[17]]]^	8:1
	H_2_SO_4_-silica gel	65	6	79^[[[13]]]^	10:1
	Bi(NO_3_)_3_.5H_2_O	60	4	83	10:1
**8**	Sulfamic acid	80	4	82^[[[17]]]^	5:1
	H_2_SO_4_-silica gel	65	6	79^[[[13]]]^	10:1
	CF_3_SO_4_-silica gel	80	48	67^[[[23]]]^	–
	Bi(NO_3_)_3_·5H_2_O	60	8	81	15:1
**9**	Sulfamic acid	80	4	81^[[[17]]]^	6:1
	H_2_SO_4_-silica gel	65	6	78^[[[13]]]^	10:1
	Bi(NO_3_)_3_·5H_2_O	60	4	76	19:1
**2**	Sulfamic acid	80	4	78^[[[17]]]^	8:1
	H_2_SO_4_-silica gel	65	6	79^[[[13]]]^	10:1
	Bi(NO_3_)_3_.5H_2_O	60	4	83	13.2:1

^a^References for reported methods

The glycosylation reaction catalyzed with bismuth nitrate yielded products with better anomeric selectivity than previously reported methods.

The research group also developed an automated flash liquid chromatography method to isolate the α- and β-anomers of glycosides using silver-nitrate-impregnated silica gel as an alternative to the classical silica gel column chromatography, which often does not perform well when trying to separate α- and β-anomers of glycosides. The peaks were detected with a UV detector and evaporative light scattering detector (ELSD) and eluted with a mixture of petroleum ether and ethyl acetate in gradient mode. All the prepared glycosides were separated to obtain pure α- and β-anomers, except the propargyl glycosides that formed a complex mixture with silver nitrate, which could not be separated. These complex mixtures were separated by flash chromatography under the same conditions [22].

### Boronic Acids as Phase-Transfer Reagents for Fischer Glycosylation Catalyzed by Camphorsulfonic Acid

Manhas et al. used boronic acids as phase-transfer reagents for Fischer glycosylation of sugars catalyzed by camphorsulfonic acid (CSA) in organic low-polarity solvents to increase the rate of the reaction, prepare functionalized glycosides, and conduct selective transformations in carbohydrate mixtures. First, the effects of boronic acid were determined with d-mannose in dichloroethane and heptane as solvents (Table 16). For the reaction, d-mannose, CSA, boronic acid, octanol and dichloroethane (DCE) or heptane, respectively, were added in a vial and purged with argon. The mixture was stirred at 80 °C for 24 h. Afterwards, the solvent was evaporated, and an aqueous solution of sodium carbonate (Na_2_CO_3_) and sorbitol was added to the mixture to split the boronic ester groups and separate the glycoside product from the boronic acid. The product was acetylated, and the anomeric ratio was determined by ^1^H NMR spectroscopy [24].

**Table 16:** Effect of boronic acids on condensation of d-mannose and *n*-octanol in low-polarity solvents [24]


No.	Solvent	R	Yields **48**^a^ (%)	Yields **49**^a^ (%)
**1**	DCE	^b^	< 5	15
**2**	DCE	Ph	60	5
**3**	Heptane	^b^	< 5	< 5
**4**	Heptane	Ph	70	< 5
**5**	Heptane	Ph^c^	40	< 5
**6**	Heptane	3,5-(CF_3_)_2_C_6_H_3_	30	< 5
**7**	Heptane	4-(MeO)C_6_H_4_	45	< 5
**8**	Heptane	Cyclohexyl	10	< 5

^a^Yields determined by ^1^H NMR spectroscopic analysis of unpurified, peracetylated reaction mixtures using 1,2,3-trimethoxybenzene as quantitative internal standard. Anomeric ratios: α:β > 20:1 for **48**, > 10:1 for **49**

^b^Results in absence of boronic acid.

^c^Using 1 equiv. PhB(OH)_2_

The reactions without boronic acids yielded low amounts of product. The main product of the reactions in presence of boronic acid was **48**α, which is unusual, since pyranosides are the products of thermodynamic control and predominant in classic Fischer glycosylation. Synthesizing furanosides with Fischer glycosylation is usually not trivial as the level of selectivity depends on the reaction conditions as well as the chosen substrate and catalyst [25], but there have been previously reported methods that used boronic esters or borate intermediates to prepare furanosides [26,27]. The highest increase in yield (70% of **48**α) was achieved using 2 equiv. phenylboronic acid relative to d-mannose in heptane. Letting methyl α-mannopyranoside react under the conditions of Table 16, entry 4 resulted in the formation of the same α-mannofuranoside at 65% yield, but at a slower rate than when using d-mannose as substrate. Several other sugars were subjected to the same reaction conditions described above, using DCE as solvent and adding phenylboronic acid [24].

Generally, the formation of boronic esters at nonanomeric OH groups was favored, except when d-glucose was used as substrate, where a furanoside-derived boronic ester was formed involving the anomeric OH group and therefore suppressing the glycosylation reaction. Utilizing *N*-acetyl-d-glucosamine instead of d-glucose prevents the participation of the anomeric OH in the formation of the boronic ester, and glycosylation could occur, leading to a mixture of furanosides and pyranosides. d-Mannose, l-rhamnose, d-lyxose, and d-ribose formed furanosides, supposedly due to the stability of boronic ester derived from *cis*-1,2-diol moieties on five-membered rings. Substrates without those moieties formed pyranosides with boronic esters involving the 4,6- or 3,4-diol groups. Utilizing the boronic ester groups of the boronic ester intermediates as protective groups, several benzoylated glycoside derivatives were also prepared. Therefore, benzoyl chloride and pyridine were added to the boronic ester intermediates before phase-switching deprotection [24].

Finally, d-mannose was mixed with d-glucose and/or d-galactose before carrying out a reaction under the conditions described above. In all cases, **48**α was formed selectively in good yield without glucose- or galactose-derived products. The authors chose the ratio of the three-component mixture to mimic the sugar content of sprucewood-derived hemicelluloses, which are a renewable chemical resource [28]. To synthesize products from carbohydrate mixtures, selective reactions, similar to the reaction presented by the mentioned research group, may be beneficial.

### Micellar Effect on Synthesis of Alkyl Glucosides by Fischer Synthesis

**Fig. 7:**
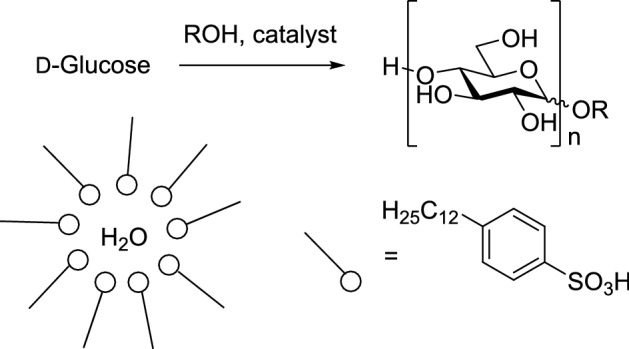
Synthesis of alkyl glucosides via the “microemulsion” route [32]

Nowicki et al. created a novel approach for Fischer glycosylation of unprotected d-glucose based on a micellar reaction system by using dodecylbenzenesulfonic acid (DBSA), which acts as both surfactant and catalyst for the reaction. A biphasic reaction system, e.g., a microemulsion, increases the reaction surface through the creation of micelles, which circumvents the poor solubility of alkyl glucosides in aliphatic alcohols [29]. The authors took advantage of the separation of reactants through micelles and the entrapment of water inside the micelles [30,31]. Figure 7 shows the proposed reaction pathway [32].

Several experiments were carried out with different aliphatic alcohols, at different temperatures, and in the presence of additional water to confirm the catalytic potential of DBSA. For the reaction, d-glucose was added to the corresponding alcohol and DBSA. The mixture was agitated at 700 rpm for 24 h at the desired temperature. The products were analyzed with high-performance liquid chromatography (HPLC), gas chromatography–mass spectrometry (GC–MS), and gel permeation chromatography (GPC). To assess the impact of APGs on glucose conversion, the commercial APG Glucopon and methanesulfonic acid (CH_3_SO_3_H) were used instead of DBSA in one experiment (Table 17) [32].

**Table 17:** Results of Fischer synthesis of alkyl glucosides in presence of surfactant-type Brønsted catalysts [32]

Entry	Alcohol	Catalyst	Reaction temp. (°C)	Water (wt.%)	Conversion (%)
**1**	1-Octanol	DBSA	60	10	96.1
**2**	1-Octanol	DBSA	70	10	66.3
**3**	1-Octanol	DBSA	80	10	55.2
**4**	1-Octanol	DBSA	60	–	79.7
**5**	1-Octanol	DBSA	70	–	80.5
**6**	1-Octanol	DBSA	80	–	99
**7**	1-Decanol	DBSA	80	–	98.2
**8**	1-Dodecanol	DBSA	80	–	97.5
**9**	1-Octanol	APG + KMS	80	–	45.7

Reaction conditions: glucose:alcohol molar ratio 1:10; DBSA 5 mol.%; 24 h; 700 rpm

Conversion of d-glucose in octanol decreased with increasing temperature when additional water was present in the reaction. The opposite effect was observed when no additional water was added, reaching 99% conversion at 80 °C. Similar d-glucose conversions are obtained with decanol (98.2%) and dodecanol (97.5%). Using an APG as surfactant and a homogeneous sulfonic acid as catalyst resulted in much lower conversion of glucose (45.7%). The authors propose that this is due to water and APGs being fully miscible and therefore not forming micelles to capture water molecules. The effect of different amounts of DBSA on sugar conversion has been tested (Table 18) [32].

**Table 18:** Effect of amount of added catalyst on glucose conversion [32]

Entry	Alcohol	Reaction temp (°C)	DBAS (mol.%)	Conversion (%)
**1**	1-Octanol	80	5	99.0
**2**	1-Octanol	80	2.5	96.4
**3**	1-Octanol	80	1	67.1

Reaction conditions: glucose:alcohol molar ratio 1:10

Lower loads of catalyst resulted in lower glucose conversion. The highest conversion (99%) was achieved with 5 mol.% DBSA.

**Fig. 8:**
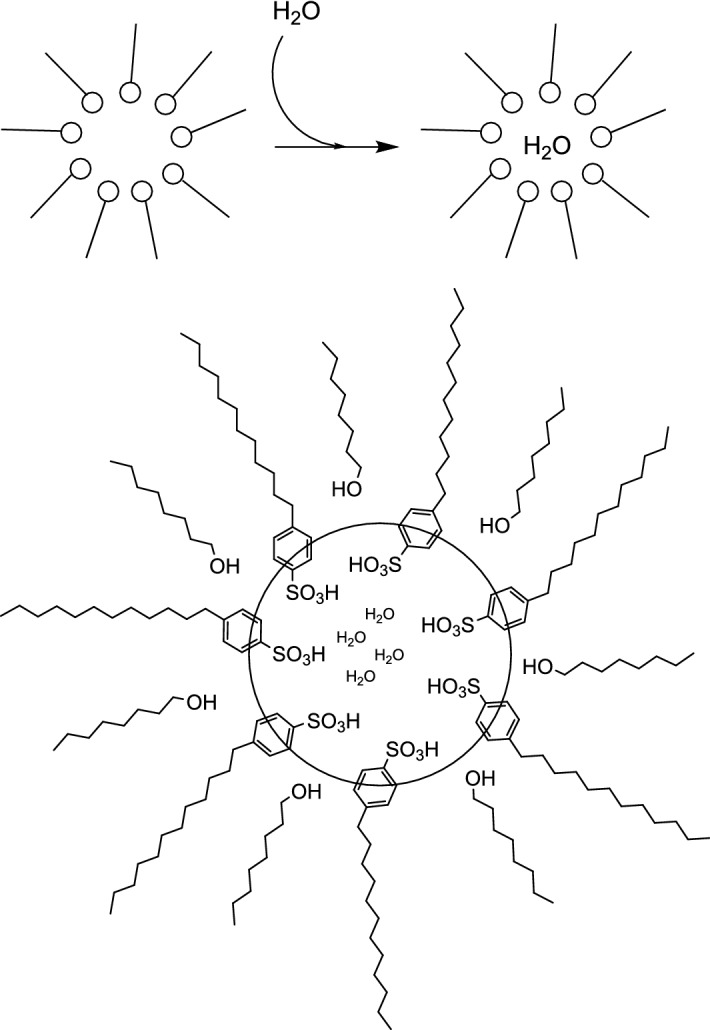
Cluster effect of water capture; proposed micelle structure (below) [32]

The water molecules resulting from the glycosylation reaction were captured in the hydrophilic interior of the cluster in dispersed phase, while the alkyl glucoside product entered the hydrophobic continuous phase (octanol). The surfactant clusters were enlarged during the reaction and formed inverse micelles with alcohol as cosurfactant (Fig. 8) [32].

This effect was also noticed by Gang et al. in solvent-free esterification in a micellar system using surfactant combined catalysts [33].

GC–MS analysis of the synthesized products of C_8_, C_10_ and C_12_ alcohols with glucose revealed that no oligomers or furanosides were formed. Only α- and β-anomers of glucopyranoside were found. The anomeric ratios are presented in Table 19 [32].

**Table 19:** α- to β-anomer ratio for selected alkyl glucosides [32]

Alkyl glucoside	Yield (%)	Anomer α (wt.%)	Anomer β (wt.%)
Oct-G	99.0	60.4	39.6
Dec-G	98.6	63.8	36.2
Laur-G	97.4	67.7	32.3

The anomeric ratios found were between 1.5:1 and 2:1, with α-glucopyranosides being the predominant anomer.

### Sulfuric Acid and Trimethylsilyl Trifluoromethanesulfonate for Synthesis of Bromoalkyl Glycosides

**Fig. 9:**
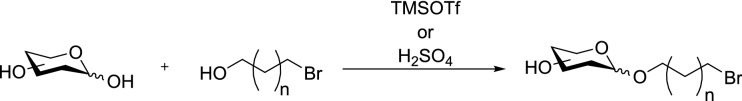
Synthetic concept and combinatorial approach toward bromoalkyl glycosides

Williams et al. used sulfuric acid and trimethylsilyl trifluoromethanesulfonate (TMSOTf) as catalysts for Fischer glycosylation of bromoalkyl glycosides synthesized from four different bromoalcohols and four different sugars. Sulfuric acid or TMSOTf was added to a suspension of monosaccharides and neat bromoalcohol, and the mixture was stirred at 80 °C for 12–16 h. The crude product was then purified by column chromatography, and the anomeric ratios were obtained by ^1^H and ^13^C NMR spectroscopy. The synthetic concept is shown in Fig. 9 [34].

Every sugar (d-glucose, *N*-acetylglucosamine, d-mannose, and d-galactose) was combined with every alcohol (bromopropanol, bromohexanol, bromooctanol, and bromodecanol), and every reaction was catalyzed with sulfuric acid or TMSOTf. The bromoalcohols were used as both solvent and reagent. The results indicated that 6 equiv. bromoalcohol relative to the monosaccharide was the lowest amount necessary to perform the glycosylation reaction. Results of the experiments are presented in Table 20 [34].

**Table 20:** Results of combinatorial approach toward synthesis of bromoalkyl glycosides [34]

Monosaccharide	Bromoalcohol n = X	Yield (%)		α:β ratio
		TMSOTf	H_2_SO_4_	
d-Glucose	1	55	65	2:1
	4	39	43	2:1
	6	27	31	2.75:1
	8	18	5	2.4:1
*N*-Acetylglucosamine	1	45	48	1:0
	4	27	20	1:0
	6	44	51	1:0
	8	32	20	1:0
d-Mannose	1	76	86	1:0
	4	53	57	1:0
	6	57	51	1:0
	8	51	31	1:0
d-Galactose	1	47^a^	47^a^	2.75:1
	4	23^a^	31^a^	
	6	16^a^	14^a^	
	8	4^a^	11^a^	

^a^No pure product isolated (purity < 80%)

The results of the reactions with d-galactose are inaccurate because column chromatography did not result in sufficiently pure product. With the other monosaccharides, yields ranging between 5% and 86% have been shown to be possible. Only pyranosides were detected by NMR analysis, and the reactions with *N*-acetylglucosamine and d-mannose resulted in pure α-anomers as products. The faster a monosaccharide dissolves in the alcohol used, the higher the yield of the product. Three combinations of monosaccharide, alcohol, and catalyst were reacted with higher equivalents of alcohol to further validate this observation (Table 21) [34].

**Table 21:** Results of three random combinations of sugar, bromoalcohol, and catalyst mixtures with increased alcohol equivalent [34]

Monosaccharide	Bromoalcohol	Catalyst	Alcohol equivalent	Yield (%)	α:β ratio
d-Glucose	Bromooctanol	TMSOTf	12	52	2.75:1
*N*-Acetylglucosamine	Bromodecanol	H_2_SO_4_	10	42	1:0
Mannose	Bromodecanol	H_2_SO_4_	10	45	1:0

Increasing alcohol equivalents resulted in large increases of yield. Excess alcohol, which was recovered in the following column chromatography, was sufficiently pure to be reused. This legitimates the use of higher amounts of excess alcohol to increase yields [34].

## Assisted Fischer Glycosylation

### Microwave-Assisted Glycosylation

The use of microwaves (MW) has become more prevalent in modern organic chemistry. Since the first reports on the use of MW, there have been a substantial number of reports about beneficial results in organic reactions run under MW conditions compared with conventional heating [35–38]. Some of these reported benefits are faster reaction times, higher yields, and higher selectivity. Additionally, MW reactions are seen as safer and more efficient than their conventional counterparts and are therefore often a great opportunity in green chemistry. Another advantage comes through the localized heating effects of microwaves, which allows reactions with less, safer, or no solvent and helps to prevent wall effects. The efficiency of reactions run under MW conditions is often dependent on the duration and intensity of the introduced MW energy [39–41]. In the context of Fischer glycosylation, there are several reports in which microwaves were used to enhance the course of the reaction (Table 22).

**Table 22:** Selected results of microwave-assisted glycosylation catalyzed by montmorillonite K10 [42]

Sugar	Alcohol	Reaction time (min.)	Yields of product^a^ (%)	α:β ratio^b^
d-Glucose	CH_3_OH	10	86	15:1
	*n*-C_4_H_9_OH	10	82	10:1
	PhCH_2_OH	10	81	9:1
		10	84	10:1
d-Galactose	CH_3_OH	10	84	11:1
	*n*-C_4_H_9_OH	10	80	10:1
	PhCH_2_OH	10	81	8:1
		10	83	9:1

Roy et al. used MW-assisted Fischer glycosylation to evaluate montmorillonite K-10 (MK10) as a reusable catalyst for Fischer glycosylation. In an initial experiment, the group reacted d-glucose with MK10 and methanol under conventional or MW conditions. Both approaches were optimized towards better α:β ratios through the study of different reaction times. For the conventional approach, complete conversion of the starting material was reached after 5 h, with the best results after 10 h with an α:β ratio of 13.3:1 and yield of 84%. The MW approach was run at 90 °C and provided a better α:β ratio of 14.6:1 as well as a slightly higher yield of 86%. The biggest difference was found in the optimal reaction time, which was only 10 min under MW conditions. To additionally validate the method of using MK10 with MW irradiation, the reaction was run with a number of different monosaccharides and alcohols, resulting in similarly good yields and anomeric selectivity. Selected results are presented in Table 23 [42].

**Table 23:** Microwave-assisted glycosylation of benzyl alcohol with d-glucose catalyzed by recycled montmorillonite K-10 [42]

Number of recycles^b^ (%)	Yield^a^	α:β
**1**	79	9:1
**2**	78	9:1
**3**	76	9:1

^a^ Isolated yield

^b^The α:β ratio was determined by 300-MHz ^1^H NMR spectroscopy

To validate MK10 as a reusable catalyst, it was reused three times for glycosylation of d-glucose with benzyl alcohol. After each reaction, the MK10 was filtrated off then activated at 110 °C, with each run resulting in similar yields and the same anomeric selectivity (Table 24).

**Table 24:** Exemplary findings for propargyl glycopyranosides (1) and 2′-azidoethyl glycopyranosides (2) [53]

No.	Product	Reaction time ( min)	Yield (%)	Ratio (α:β)
**1**	**39**	30	85	10:1
**2**		30	90	20:1

The group concluded that the use of MK10 in combination with MW irradiation represents a fast, inexpensive, and ecofriendly method for Fischer glycosylation [42].

**Fig. 10:**
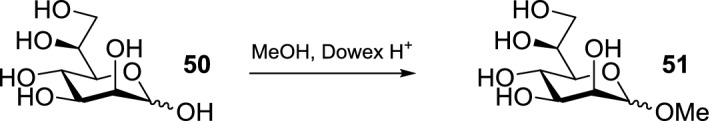
Glycosylation of l-*glycero*-d-manno heptose to l-*glycero*-α/β-d-*manno*-heptopyranoside

In a study with the aim of generating simple model glycosides for lectin binding studies, Artner et al. synthesized l-*glycero*-α-d-*manno*-heptopyranoside through Fischer glycosylation of heptopyranose with methanol in presence of Dowex 50 H^+^ cation-exchange resin, first under classical conditions at 60 °C and second under microwave irradiation (Fig. 10) [43].

**Fig. 11:**
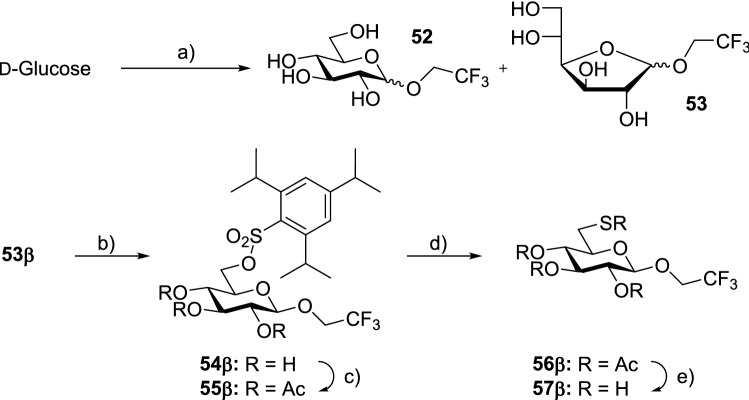
Reagents and conditions: **a** TFE, H^+^ cat., 160 °C MW, 10 min, 51% **b** 2,4,6-trisisopropylbenzene-sulfonyl chloride, pyridine, 71% **c** Ac_2_O, pyridine, quant. **d** N_2_, KSAc, DMF, 78 °C, 20 h, 92% **e** N_2_, NaOMe, MeOH, 71%

Comparatively, the conventional procedure afforded a 81% combined yield with an α-to-β ratio of 7:1 of **51** after several hours of reaction. On the other hand, microwave irradiation at 100 °C for 25 min resulted in a higher yield of 94% and an α-to-β ratio of 10:1, with the mixture additionally containing 4% of anomeric furanosides. The products were later acetylated for purification (Fig. 11) [43].

In another MW approach for Fischer glycosylation, Fröhlich et al. described the synthesis of a glucopyranoside containing a trifluoromethyl- and a thiol group for ^19^F NMR spectroscopy on cysteine in proteins, the ^19^F being necessary for NMR spectroscopy and the thiol group for selective binding to the cysteine. Only the attachment of 2,2,2-trifluoroethanol (TFE) was achieved through MW-assisted Fischer glycosylation in this research and is thus the only reaction step considered in detail here (Fig. 14). First attempts to react TFE with d-glucose under classic Fischer glycosylation conditions were unsuccessful, so the group opted for MW-assisted Fischer glycosylation under increased pressure. The group used d-glucose (1.35 g, 7.5 mmol) dissolved in TFE (15 mL) with acetic chloride (10 μL, 0.14 mmol) in a microwave reactor (Anton Paar) for 10 min at 160 °C with stirring at 600 rpm (Fig. 11a). This resulted in a conversion to the expected trifluoroethylglucoside with the pyranosides constituting the major product (75%), in which the α-anomer was the predominant anomer at 60%. In subsequent reaction steps, the group was unable to isolate the α-anomer, thus the β-anomer was used for subsequent steps, meaning the conversion of the primary hydroxy group with 2,4,6-trisisopropyl-benzenesulfonic chloride (Fig. 14b). For the later displacement of leaving group on carbon 6, the product had to be acetylated and later deprotected **57**β (Fig. 14c) [44].

Ceron-Camacho et al. conducted a study to directly compare Fischer glycosylation assisted by MW versus conventional heating (CH) by synthesizing APGs from d-glucose and d-mannose with alkyl alcohols of different length. Under MW conditions, the reaction parameters were optimized through a model reaction between glucose and 1-dodecanol. The best results were obtained with a 3 min reaction time at 70 °C and 5 W of power. The same model reaction was run under CH in an oil bath at 70 °C, as well. To provide better comparability, both reactions were run in the same sealed vessel system. The group found that the MW reaction resulted in a lower conversion of glucose at 8.5% residual glucose compared with 1.4% residual glucose under CH. Also a slightly higher degree of polymerization (DP) was found under CH (DP = 1.8) compared with MW (DP = 1.6). Note that the group compared the results of both approaches in a time frame of 3 min at 70 °C, but the conventional heating took much longer at 650 s compared with 80 s for the MW to reach said temperature. This results in a higher overall reaction time for the CH [45].

In a further study, Aronow et al. questioned the scalability of MW-assisted Fischer glycosylation and thus investigated the possibility of translation into a continuous flow process, directly comparing it with the MW-mediated process. For this, see Sect. 4 [46].

### Ultrasonic-Assisted Glycosylation

In the late 1920s, Loomis was the first to describe the benefits of using ultrasound in chemistry. He demonstrated an accelerating effect on the hydrolysis of dimethyl sulfate when using sound wave radiation [47]. In recent years, with the need for greener and safer approaches to organic chemistry, the use of ultrasonic waves has become a promising technique. This approach has since been incorporated into a wide variety of reactions, forming its own discipline called sonochemistry. Most sonochemical reactions are run at frequencies from 20 to 100 kHz and are driven by the kinetic energy caused through cavitation [48,49]. Cavitation describes the periodic growth and compression of air microbubbles in a liquid phase. This subsequently results in the collapse of the microbubbles, causing a rise of pressure and temperature that has been calculated to be up to 1000 atm and 500 K, respectively [50]. This results in a series of chemical and physical effects that can benefit a chemical reaction.

Amaniampong et al. reported on the possible usage of ultrasonic-assisted, catalyst-free Fischer glycosylation for production of APGs. In the current state of the art, APGs are produced by traditional acid-catalyzed Fischer glycosylation [51]. One limitation of this procedure is the attainable DP of only 1.1–1.5 glucose units per alkyl chain. A higher DP of around 2–8 would be highly beneficial for the use of APGs by enabling a number of beneficial effects [52].

In 2011, Shaik et al. proposed a procedure for improved synthesis of azidoethyl and propargyl glycopyranosides through Fischer glycosylation using H_2_SO_4_-silica (prepared according to [13]) as catalyst in addition to ultrasound radiation. The free sugars used were *N*-acetyl-d-glucosamine, *N*-acetyl-d-galactosamine, d-glucose, d-galactose, d-mannose, l-fucose, and lactose. They were glycosylated with propargyl alcohol or 2-azidoethanol at 40 °C with H_2_SO_4_-silica under ultrasonication, which was run until the starting materials disappeared. Selected results are presented in Table 24. The group noted that the success of the procedure depends on the solubility of the sugar in the used alcohol. This was seen as a possible explanation for the longer reaction time with lactose, where there was no significant improvement in reaction time compared with already existing methodology, but better yields were observed. Overall reaction times ranged from 15 min to 2 h, with the exception of lactose of 12 h. Yields also saw an improvement compared with reported procedures, ranging from 70% to 98%, and α-glycopyranosides were found to be the most common product. The group concluded that ultrasonic-assisted Fischer glycosylation is an efficient tool for glycosylation of significant monosaccharides because of its better yields and reaction times compared with traditional, Fischer glycosylation [53].

To test the usability of ultrasonic-assisted, catalyst-free Fischer glycosylation, the group subjected the sugars mannose, glucose, and xylose to ultrasonic irradiation at 550 kHz for 3 h in a solution of methanol. The impact of experimental parameters was examined by using mannose under an array of different conditions. The parameters that were modified included temperature (40 °C, 20 °C, and 0 °C at 40 wt.% man. under air), wt.% mannose (40% and 80% at 40 °C under air), and type of gas (air, Ar, and O_2_ at 40 °C with 40 wt.% mannose) (Table 25) [54].

**Table 25:** Impact of experimental parameters on high-frequency ultrasound efficiency^a^ [54]

No.	Temp. (°C)	Mannose conc. (wt.%)	Gas	Conv. mannose (%)	Yield 1,6-anhydro-mannose (%)	Yield Me-Mannoside (%)	Yield Me-alkyl-polymannoside (DP ≥ 2) (%)^b^
**1**	40	40	Air	81	2	37	42
**2**	20	40	Air	58	1	26	31
**3**	0	40	Air	6	0	2	4
**4**	40	40	Ar	70	1	35	34
**5**	40	40	O_2_	50	0	21	29
**6**	40	80	Air	73	1	6	66

^a^Ultrasonic irradiation at 550 kHz for 3 h (0.44 W L^−1^)

^b^Also includes terminal free oligomannosides

It is notable that varying the temperature had no effect on reaction selectivity and resulted in a decrease in the percentage of converted mannose. The usage of Ar and O_2_ gases similarly reduced the conversion, to 70% and 80%. Running the reaction at 40 wt.% under air at 40 °C led to an 81% conversion with yield of 37% methyl mannosides (MeMan) and 42% Me-alkylpolymannoside with a DP higher than 2. The reaction delivered a DP range of 1–12 with an average DP of 7. The pyranoside-to-furanoside ratio was found to be 7:1, with the product consisting of 58% monosaccharides (mannose, methyl-mannosides, 1,6-anhydromannose), 12% disaccharides (Me(Man)_2_, Man_2_), and 30% APGs with DPs higher 3. Furthermore, the group was able to increase the concentration of mannose to 80 wt.% (Table 25, entry 6), resulting in a mannose conversion of 73% with APGs with DPs from 2–7 at 66% yield. The space–time yield in this scenario was found to an unprecedented 876 kg m^−3^ h^−1^ [54].

To further investigate the usability of ultrasound technology, glucose and xylose were also assessed under standard conditions (550 kHz under methanol for 3 h/6 h). After 3 h, 40% conversion of glucose was reached. By increasing the reaction time to 6 h, the conversion could be improved to 82%. For xylose, the conversion increased from an initial 82% at 3 h to 86% at 6 h. Analysis of the glucose reaction indicated an average DP of 2, with glucopyranosides being the major component at 99%. With xylose, an average DP of 3 was found with an xylopyranoside-to-xylofuranoside ratio of 1:10. Additionally, other alcohols (ethanol, *n*-propanol, and *n*-butanol) were tested, showing conversions of 70%, 66%, and 87%, respectively. Also, data obtained from matrix-assisted laser desorption–ionization (MALDI)-time of flight (TOF) analysis of the mannosidic-derived APGs revealed 1,6-anhydromannose and its mannosylated derivatives as possible key intermediates in this process.

The group concluded that high-frequency ultrasound allows selective activation (with no product having a methoxy group at a position other than the anomeric center) of the anomeric position of the analyzed sugars without the use of a catalyst as in traditional Fischer glycosylation [54].

## Fischer Glycosylation in Reactors

**Fig. 12:**
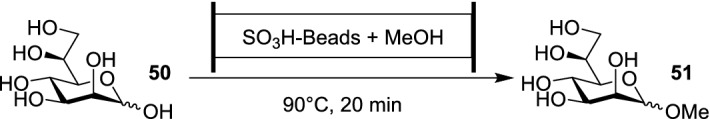
Fischer glycosylation of l-*glycero*-d-*manno*-heptopyranose in a flow reactor with methanol and sulfonic acid beads

One limiting factor of MW-assisted glycosylation is its poor scalability [55]. Aronow et al. proposed a transition from microwave batch to continuous flow processing for the synthesis of methyl glycosides. Preceding their proposition to use flow reactors as a possible solution to the scalability issue of MW, the group had already successfully used a continuous flow process in their report on the synthesis of l-*glycero*-d-*manno*-heptopyranose peracetate, a major constituent in Gram-negative bacteria [56]. In their proposed process, the group used a continuous flow process to glycosylate l-*glycero*-d-*manno*-heptopyranose with MeOH and sulfonic acid beads. Optimal results were found with temperatures of 90–100 °C and residence times from 10 to 25 min. The reaction resulted in a α:β pyranoside ratio of 7:1 with approximately 3.5% of α-furanoside. The methyl heptosides were then converted to the desired product in subsequent reaction steps (Fig. 12) [56].

Building on this successful use of flow chemistry, Aronow et al. conducted a comprehensive study comparing microwave and flow approaches for Fischer glycosylation. The group compared the glycosylation reactions of different sugars with methanol under the same reaction conditions in a MW-assisted approach as wells as in a flow reactor. The flow process was optimized by varying the temperature and residence time using the glycosylation reaction of d-mannose and methanol. Optimal conditions were found at 120 °C with a residence time of 4 min. These parameters were then also applied to all following MW reactions. Throughout the following reactions, high consistency between MW and flow conditions was obtained. Exemplary results of this study are presented in Table 26. Note that, for sugars that are unsolvable in MeOH, minimal amounts of water were added under flow conditions [46].

**Table 26:** Exemplary findings of comparisons of Fischer glycosylation between continuous flow and microwave conditions [46]

Entry	Sugar	Mode	H_2_O add. (%)^a^	Isomer ratio (%)^b^				s.m. (%)^b^
				Pyranoside		Furanoside		
				α	β	α	β	
**1**	d-Mannose	Flow	–	85	8	5	2	< 1
**2**		MW	–	81	7	10	3	< 1
**3**	d-Glucose	Flow	7.5	67	31	1	1	3
**4**		MW	–	64	31	2	3	3
**5**	d-Galactose	Flow	35	51	27	6	16	3
**6**		MW	–	39	20	11	30	1

*Flow* flow reactor, *MW* microwave

^a^Refers to % (v/v) water per solution

^b^Determined by ^1^H NMR spectroscopy

Scalability of the approach was successfully demonstrated through the formation of methyl mannosides under these standard conditions, generating a throughput of 1.2 g/h of crude product in a 10 h run. Throughout the run, a consistent α:β ratio was observed, which confirmed stable catalyst activity. After recrystallization of the crude product, the group was able to yield 9.4 g of the desired product, almost four times the mass of catalyst used [46].

Masui et al. reported on a procedure for kinetically controlled Fischer glycosylation under flow conditions for synthesis of furanosides. The approach was first tested with glucose and methanol and β-hydroxy-substituted sulfonic acid functionalized silica (HO-SAS) as catalyst. Good conditions were found to be 80 °C with 5 min residence time, resulting in 71% yield of furanosides (α:β ratio of 35:65) and 100 °C with 1 min residence time resulting in 70% yield of furanosides (α:β ratio of 43:57). Other catalysts, HCl and TsOH, were also tested, but sHO-SAS was found to be the better choice for this approach. Scalabillity and reusabillity of HO-SAS were also tested with positive results. The kinetically controlled flow approach was then applied and optimized on three additional saccharides (mannose, galactose, and *N*-acetylglucosamine). *N*-Acetylglucosamine was the only sugar for which only pyranosides were formed while no furanosides could be obtained. For mannose, the optimal conditions were found to be 100 °C with residence time of 30 s, resulting in a yield of 67% with an α:β ratio of 76:24. The optimal residence time for galactose was found to be much higher at 15 min with a temperature of 60 °C, resulting in 86% yield with an α:β ratio of 26:74 [57].

**Fig. 13:**
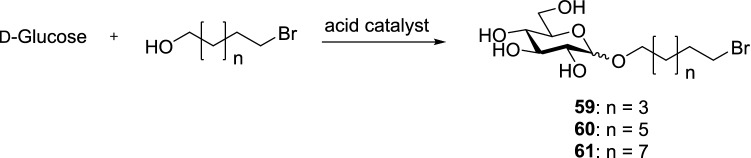
Visualization of Fischer glycosylation with d-glucose and longer bromoalcohols

Another approach to overcome the scalability problem of MW-assisted glycosylation is the use of microreactor technology, which can be described as a scale-down technique using a device with submillimeter dimensions in which chemical reactions are performed continuously [58]. Jung et al. developed a method for Fischer glycosylation of bromoalkyl glycosides in a microreactor, which offers excellent heat and mass transfer to optimize the yield and selectivity of a reaction in a time- and material-saving manner. Microreactors are also a safer alternative for MW Fischer glycosylation, where a headspace is created through flammable alcohols, which represents an explosion hazard. Figure 13 shows the synthesis of bromoalkyl glucosides from d-glucose and bromoalcohols promoted by an acidic catalyst [59].

**Fig. 14:**
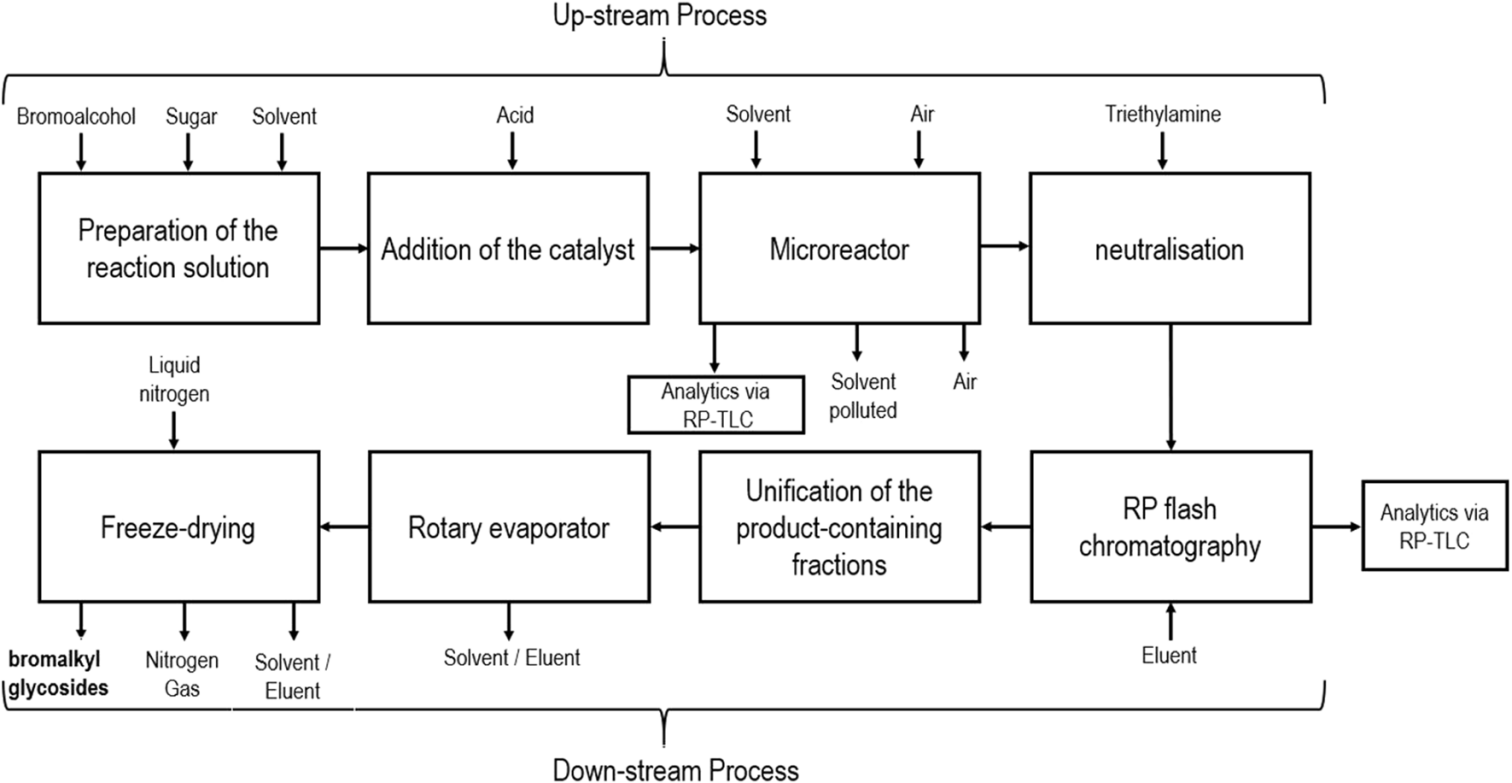
Schematic of overall process for obtaining bromoalkyl glycosides from bromoalcohols and reducing sugars

Figure 14 shows the process for obtaining the bromoalkyl glucosides. Dimethyl sulfoxide ((CH_3_)_2_SO) (DMSO) was used for conditioning of the microreactor and as a solvent. After stopping the reaction by neutralizing the acidic catalyst with triethylamine (Et_3_N), the reactor was dried with air. The crude product was purified first with column chromatography, and after combining the fractions containing bromoalkyl glucosides, organic solvents were removed under reduced pressure to allow further refinement of the product with lyophilization. Anomeric ratios were detected with ^1^H and ^13^C NMR spectroscopy [59].

The reaction was carried out with alcohols of different length and different catalysts at 75 °C, 90 °C, or 120 °C, and purification of the product was carried out by TLC, liquid–liquid extraction, and normal-phase (NP) and reverse-phase (RP) flash chromatography.

Using 2-bromoethanol or 3-bromo-1-propanol in the reaction did not produce the desired bromoalkyl glucosides. The authors assume that these short alcohols are too reactive due to the higher electrophilicity of the carbon bearing the bromide, which leads to polymeric ethyleneglycol or propyleneglycol side products. It is further discussed that potentially produced glycosidic products can react further with these side products in the microreactor or with the silica during the NP chromatographic work-up. Experiment number 3 and 4 support this assumption. In experiment 5, RP flash chromatography was used, but the reaction was carried out at 90 °C, which indicates that higher temperatures were needed to produce bromoalkyl glucosides. No product was isolated in the experiments with *n*-octanol when NP flash chromatography or liquid–liquid extraction was used to purify the product; only RP flash chromatography led to successful isolation of the product. Sulfuric acid (H_2_SO_4_), trifluoroacetic acid (CF_3_CO_2_H), and TMSOTf were compared in their catalytic activity with TLC. Using similar amount of reaction solution and *n*-octanol resulted in spots with the same RF value, but the spots were more intense when TMSOTf was used as catalyst. The authors assume that product was produced with all catalysts, but TMSOTf produced the highest yield. In the experiments where bromoalkyl glucosides were detected, TMSOTf was used as catalyst and RP flash chromatography was used as purification method. Yields between 24% and 40% were achieved with 6-bromo-1-hexanol, 8-bromo-1-octanol, and 10-bromo-1-decanol, and corresponding dibromides were isolated, which very likely are impurities in the bromoalcohols. Compared with the yields obtained by Williams et al. [34], an increase of 150% in experiments with 8-bromo-1-octanol and an increase of over 180% in experiments with 10-bromo-1-decanol were achieved. The research group points out that loss of reaction solution that occurred while handling the microreactor during the reaction is not included in the yield calculations. Since the volume of reaction solution was only 3–4 mL, even small losses of solution could significantly influence the product yield, which may be the reason for the lower yield in experiments with 6-bromo-1-hexanol compared with Williams et al. [34]. Higher yields could be expected when more reaction solution is used, since the loss of a few hundred microliters would not impact the yield calculation as much [59].

## Outlook and Conclusions

Several advances have been made to improve the classic Fischer glycosylation for production of glycosides. New catalysts have been selected for not only their ability to increase the efficiency of their respective reaction but also being environment friendly, which is an important factor for discoveries in modern times. Protection of non-anomeric OH-groups was not necessary with most catalysts, thereby reducing reaction and purification steps. Creating a biphasic solution to capture water molecules (that result as byproduct in Fischer glycosylation) in micelles is a novel method to improve the poor solubility of alkyl glucosides in alcohol and could be explored further in the future with other monosaccharides. Microwave- and ultrasonic-assisted reactions are promising instrumental methods for Fischer glycosylation. Sonochemistry allows for much longer alkyl chains in alkyl glycosides, while microwave-assisted reactions resolve significantly faster than the standard procedure, often needing only minutes instead of hour-long reactions while retaining good yields and selectivity. However, to date, microwave-assisted glycosylation remains impractical for industrial use as it is hard to scale up. Fischer glycosylation in micro- and continuous flow reactors are relatively new processes that promise good scalability and efficient optimization of reactions. These new approaches provide good alternatives to inefficient, time-consuming, and polluting procedures for the production of glycosides.

## Data Availability

The datasets generated during and/or analyzed during the current study are available from the corresponding author on reasonable request.

## Code availability

Code availability is not applicable.
